# Resorbable magnesium metal membrane for sinus lift procedures: a case series

**DOI:** 10.1186/s12903-023-03695-4

**Published:** 2023-12-14

**Authors:** Akiva Elad, Luka Pul, Patrick Rider, Svenja Rogge, Frank Witte, Dražen Tadić, Eitan Mijiritsky, Željka Perić Kačarević, Larissa Steigmann

**Affiliations:** 1 Private Practice, Tel Aviv, Israel; 2 Department of Dental Medicine, Faculty of Dental Medicine and Health Osijek, J.J. Strossmayer University of Osijek, Crkvena 21, 31 000 Osijek, Croatia; 3 Botiss Biomaterials GmbH, 15806 Zossen, Germany; 4 Department of Prosthodontics, Geriatric Dentistry and Craniomandibular Disorders, Charité–Universitätsmedizin Berlin, Aßmannshauser Straße, 4–6, 14197 Berlin, Germany; 5 Department of Head and Neck and Maxillofacial Surgery, Tel-Aviv Sourasky Medical Center, The Sackler Faculty of Medicine, Tel-Aviv University, 6139001 Tel Aviv, Israel; 6 Department of Anatomy, Histology, Embryology, Pathology Anatomy and Pathology Histology, Faculty of Dental Medicine and Health Osijek, J.J. Strossmayer University of Osijek, Crkvena 21, 31 000 Osijek, Croatia; 7 Department of Oral Medicine, Infection, and Immunity, Division of Periodontology, Harvard School of Dental Medicine, Boston, MA USA

**Keywords:** NOVAMag membrane, Resorbable metal, Sinus lift augmentation, Schneiderian membrane, Regenerative dentistry, Barrier membranes

## Abstract

**Background:**

The purpose of this case series was to demonstrate the use of a magnesium membrane for repairing the perforated membrane in both direct and indirect approaches, as well as its application in instances where there has been a tear of the Schneiderian membrane.

**Case presentation:**

The case series included four individual cases, each demonstrating the application of a magnesium membrane followed by bone augmentation using a mixture of xenograft and allograft material in the sinus cavity. In the first three cases, rupture of Schneiderian membrane occurred as a result of tooth extraction, positioning of the dental implant, or as a complication during the procedure. In the fourth case, Schneiderian membrane was perforated as a result of the need to aspirate a polyp in the maxillary sinus. In case one, 10 mm of newly formed bone is visible four months after graft placement. Other cases showed between 15 and 20 mm of newly formed alveolar bone. No residual magnesium membrane was seen on clinical inspection. The vertical and horizontal augmentations proved stable and the dental implants were placed in the previously grafted sites.

**Conclusion:**

Within the limitations of this case series, postoperative clinical examination, and panoramic and CBCT images demonstrated that resorbable magnesium membrane is a viable material for sinus lift and Schneiderian membrane repair. The case series showed successful healing and formation of new alveolar bone with separation of the oral cavity and maxillary sinus in four patients.

## Background

The loss of teeth in the maxillary arch leads to physiological, anatomical and functional changes, including the loss of vertical and horizontal dimensions of the oral cavity. Post tooth extraction, the functionality and subsequent pressure on the alveolar bone which stimulates the process of remineralization in healthy dentition, is lost. This manifests over the years as the resorption of the alveolar ridge with the surrounding hard and soft tissue.

Dental implantology has proposed new approaches for prosthetic restoration of patients in whom the maxillary arch has been edentulous for a long period of time. In this situation, the maxillary sinus is pneumatized and the alveolar ridge is atrophied. Therefore, for implant placement, sinus floor elevation and the use of a bone graft substitute are required [1–4]. The conventional time for placement of dental implants after bone regeneration procedures is six months postoperative according to the literature [5, 6], but there are recent references that assume a shorter healing time of three to five months and is dependent on the indication and the graft materials used [7, 8]. The retrospective study by Park et al. [9] showed interesting results describing the influence of residual bone height and perforation of the sinus membrane on the success rate of implants. In cases where the residual bone height was less than 3 mm, the survival rate was significantly lower. The study claims that there was no statistically significant difference in survival rate between cases with and without perforation of the sinus membrane, although membrane perforated cases had a higher incidence of sinusitis.

In 1998. professor P. Brånemark proposed a new solution for the rehabilitation of patients with missing bone structure by inserting zygomatic implants. Over the years, the technique was changed from intrasinusal to extrasinusal, making the procedure less invasive [10]. However, serious complications can develop with zygomatic implants such as a lack of osseointegration of the coronal part of the implant and the formation of oroantral fistula and maxillary sinusitis [11, 12].

It has been shown that in 40% of cases, the roots of the maxillary first and second molars are in contact with the maxillary sinus [13]. Hence, extractions can create a communication between the maxillary sinus and the oral cavity. In such situations, oroantral communication can spontaneously heal or needs to be sealed with resorbable membranes [14].

Although the maxillary sinus lift procedure has been thoroughly researched over the past decades and provides predictable outcomes, complications are not uncommon. The sinus lift procedure was first described by Tatum in the 1970s [15]. It involved a combination of incisions: a crestal incision at the alveolar ridge with two vertical incisions mesial and distally. This incision design allows for an elevation of a buccal flap which exposes the lateral bone wall of the maxillary sinus. Following, access to the Schneiderian membrane and sinus cavity is ensured by a trapdoor osteotomy on the lateral wall. By elevating the sinus membrane in the cranial direction, space can be created for the graft material [15, 16].

A less invasive technique (indirect/vertical sinus lift) does not require an external approach and lateral wall access. After gradual widening by the osteotomy, the sinus floor is accessed with an osteotome and followed by careful elevation of the Schneiderian membrane. If necessary, graft material is placed between lifting the membrane [17].

The most common complication of a maxillary sinus lift is Schneiderian membrane perforation (SMP) [18]. Other complications include postoperative infection and persistent bleeding [19]. Al-Dajani et al. [18] reported that membrane perforation occurs in 3.6% to 41.8% of the cases, with decreased membrane thickness and sinus septa increasing the risk of perforation. Several therapeutic approaches to repair of the Schneiderian membrane have been proposed: covering it with a resorbable membrane, carefully suturing the Schneiderian membrane or folding the sinus membrane onto itself to close the gap [20]. However, a complete tear of the Schneiderian membrane will often result in the discontinuation of surgery and a delay to the procedure of between 3 – 6 months [21, 22].

If the Schneiderian membrane is perforated, common postoperative complications such as infection and sinusitis may occur. It is crucial to preserve and maintain the Schneiderian membrane’s barrier function in order to prevent bacterial invasion and the risk of infection. However, even if a resorbable collagen membrane is used to close the Schneiderian membrane, there are reports of a higher prevalence (31.4%) of sinusitis compared to cases in which the membrane was not perforated during sinus lift (6.2%) [23].

Associations have also been made between membrane perforation and graft failure, however there are conflicting reports regarding this relationship [24–26]. In a review on complications of maxillary sinus augmentations by Kim et al. [27], it was determined that the implant survival rate is only affected with perforations larger than 5 mm. A correlation between thicker sinus membranes and perforations due to thicker membrane pathosis, poorer vascularity and elasticity in contrast to thin membranes was described by Park et. al. [28]. Furthermore, in their study, implants placed in patients with unrepaired Schneiderian membrane perforation had the same survival rate as non-perforated membranes. Nevertheless, without an intact sinus barrier, there is the risk of graft dislodgement into the sinus, where it can obstruct the ostium and prevent drainage. Thus, a resorbable barrier membrane can be utilized to cover the perforated area/perforation, which not only reduces the risk of infection but also prevents the displacement of the bone graft. Primarily, a collagen membrane is used for such repairs, however, for larger perforations, it is not possible to suture the collagen membrane to the Schneiderian membrane. Additionally, if the Schneiderian membrane is too thin, it can tear during suturing. The mechanical support provided by the collagen membranes might also be insufficient for large perforations. Therefore, it has previously been recommended that collagen membranes should only be used for perforations less than 10 mm [19].

To overcome this issue associated with the repair of large perforations using a collagen membrane, other treatment methods have been proposed, such as the use of freeze dried human lamellar bone sheets [25]. This material can provide a rigid support to the augmentation; however, it is not easily adapted to the anatomy.

An alternative solution could be the use of a recently developed magnesium membrane [29, 30], especially for instances where there has been a complete tear of the Schneiderian membrane. The magnesium membrane is reportedly much stronger than that of collagen membranes [29]. Moreover, the material is completely resorbable, eliminating the need of membrane removal after the healing period is completed.

In a series of four cases, the first implementation of the magnesium membrane for sinus lift augmentations is presented, including three cases with a perforation of the Schneiderian membrane.

## Materials and methods

### Patients

Three female and one male patient participated in this case study. Their ages ranged from 35 to 61 years, with the 61 being the oldest. All patients exhibited good general health on preoperative clinical exam. All four patients were indicated for sinus lift procedure followed by dental implant placement immediately or several months post-operative.

### Surgical procedure

#### Tooth extractions and sinus lift procedure

In each case, the teeth or dental implants were carefully extracted, followed by thorough debridement. All patients experienced severe loss of maxillary alveolar bone or peri-implantitis, which was diagnosed by analysis of panoramic images and cone beam computed tomography (CBCT) data. After extraction of the teeth or implants, in three cases the Schneiderian membrane ruptured due to the anatomy of the teeth, position of the implants or as complication during sinus lift procedure. This case series consists of four individual cases without control group, demonstrating the possibility of treatment using a magnesium membrane. In the first two cases after molar extraction, there was direct communication with the maxillary sinus. They were treated with an indirect (vertical) approach, while the other two cases were treated with a direct or open sinus lift, in which the buccal wall was surgically opened to provide easier access to the floor of the maxillary sinus.

#### Application of magnesium membrane

Once the desired access to Schneiderian membrane was established, a sterile magnesium membrane (©® membrane, botiss biomaterials GmbH, Germany) with initial dimensions of 30 × 40 mm and thickness of 140 µm was prepared. Using NOVAMag® scissors, the membrane was cut to the required size, whilst ensuring that there were no sharp edges that could perforate the soft tissue. Where the rim of the membrane had a raised edge after©g, the NOVAMag® sculptor was used to flatten the edge. The membrane could then be bent to the necessary shape to fit over the defect.

#### Positioning of bone augmentation material

In each case the defect was filled with a combination allogeneic bone granules (maxgraft®, botiss biomaterials GmbH) and xenogeneic bone granules (cerabone®, botiss biomaterials GmbH) and a second©ium membrane (NOVAMag® membrane, botiss bio-materials GmbH, Germany) was placed over the augmentation and the flaps were sutured. The second membrane placed over the lateral window was used to prevent the displacement of graft material and reduce the risk of associated post-operative complications [31].

#### Insertion of dental implants

**Fig. 1:**
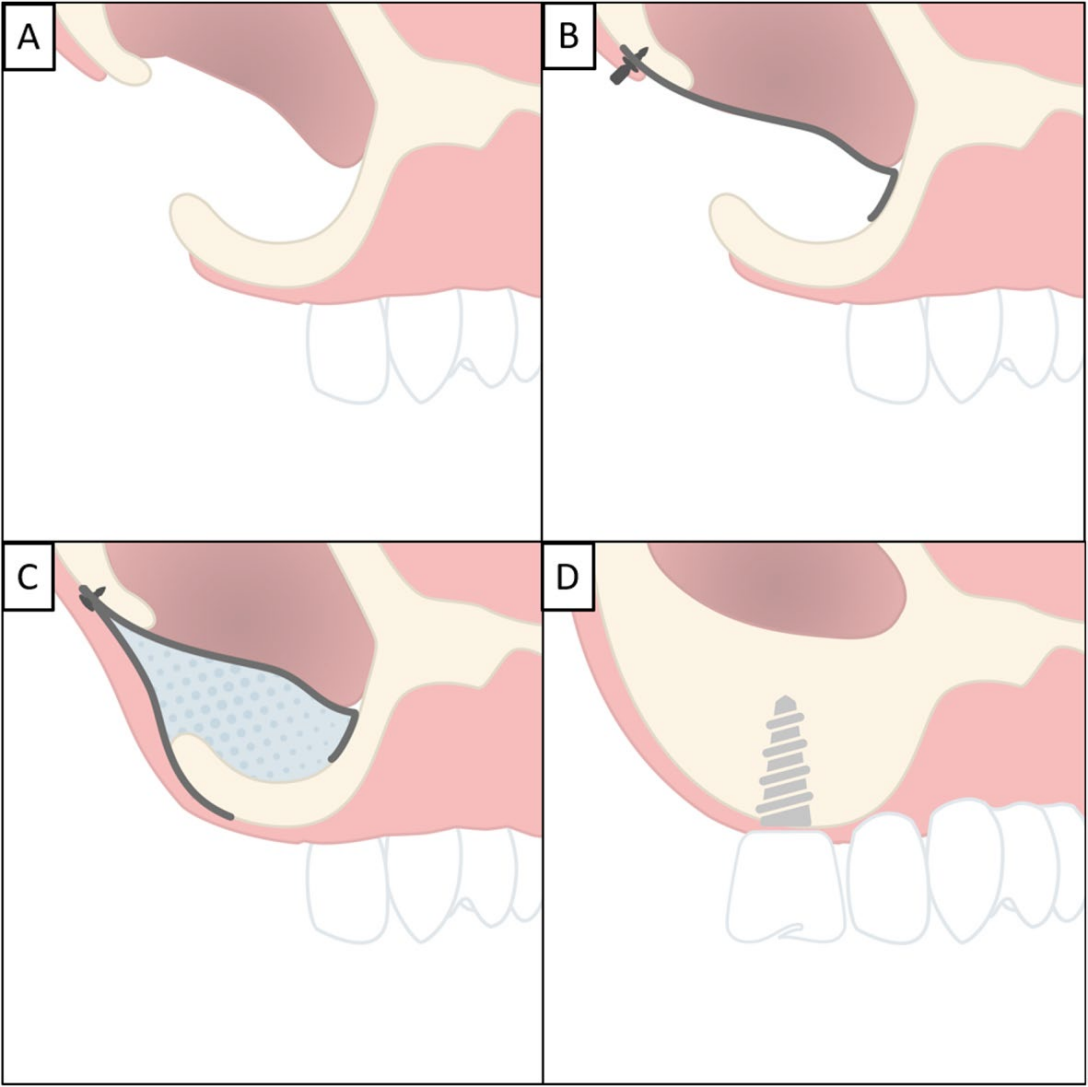
Sinus lift procedure according to case 3. **A** During extraction of maxillary teeth or as an intraoperative complication, rupture of Schneiderian membrane results in a direct communication between the maxillary sinus and the oral cavity. **B** The resorbable membrane is placed in a single layer and bent to form the floor of the maxillary sinus, which rests on the surrounding alveolar ridge. After placement, the surgeon can choose to fixate the membrane with a titanium or magnesium fixation screws (NOVAMag® fixation screw XS, botiss biomaterials GmbH). **C** After correct placement and fixation of the resorbable membrane, graft material is inserted to stimulate the formation of new bone and replace the lost alveolar bone. In the final step, the graft material is covered with another magnesium membrane or a collagen membrane to separate the augmentation from the surrounding soft tissue and prevent ingrowth that could interfere with the bone remineralization process. **D** After six months, the membranes are resorbed and new bone has formed at the augmentation site. The dental implant can be placed in the hard tissue providing the initial stability

Four to five months later, the flap was opened and implants were placed in the augmented bone (Fig. 1.). In the included cases, long implants were use (> 8 mm), however it is also possible to use short implants, with the decision based upon the discretion of the practicing clinician. Post-operatively, patients were instructed to rinse twice daily with chlorhexidine solution for 2 weeks.

## Case presentation

### Case 1

The patient was a 35-year-old female in good general health. The patient presented with teeth 16 and 17. The CBCT scan showed a severe periapical lesion on tooth 17 that extended into the sinus and completely destroyed the floor of the right maxillary sinus.

**Fig. 2:**
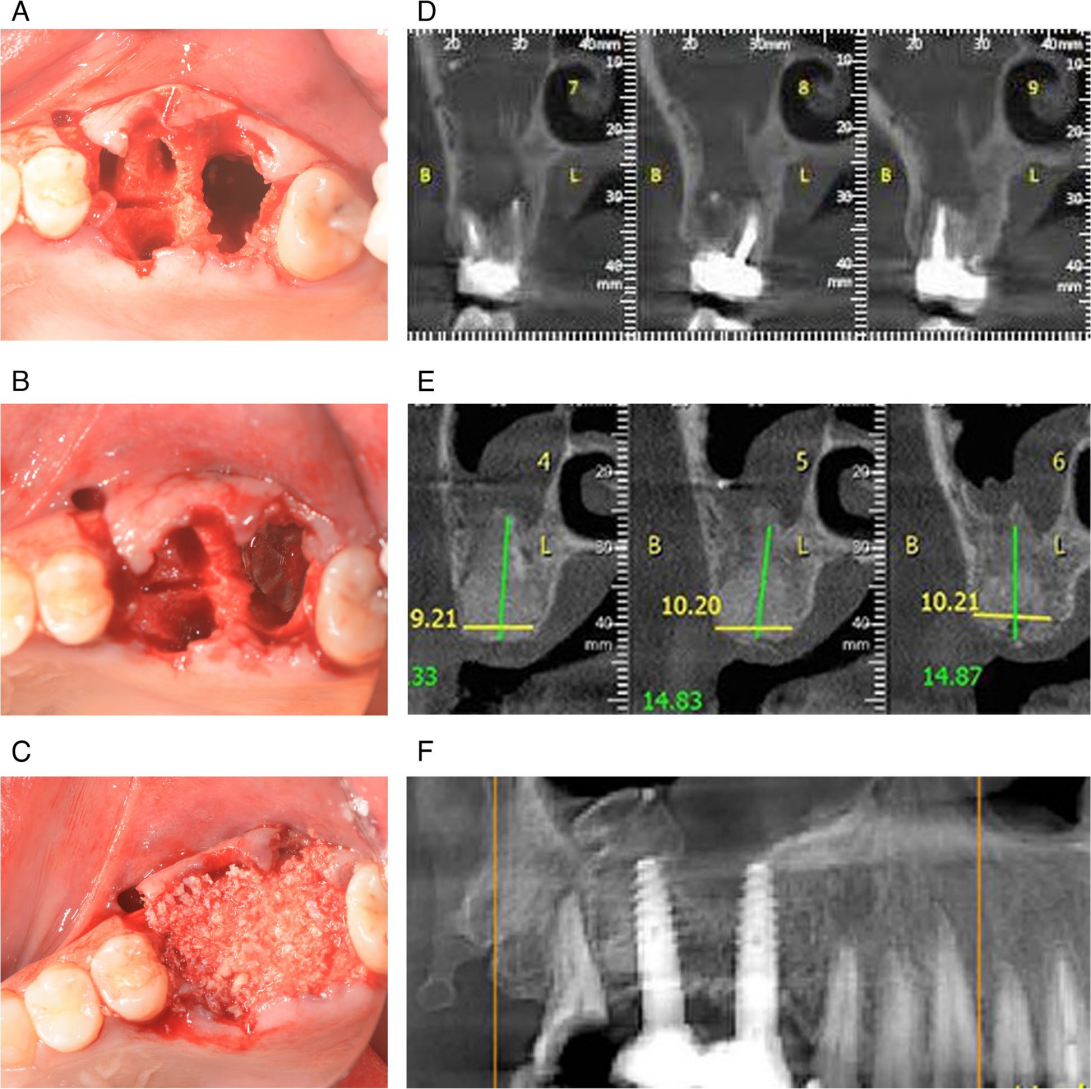
**A** After serial extraction of teeth 16 and 17, oroantral communication at the site of the maxillary second molar was clinically noted and diagnosed. **B** The magnesium membrane (black arrow) was folded and placed in a way that the communication was closed. **C** Allograft and xenograft granules were used for augmentation and a second magnesium membrane was placed over the augmentation. **D** Coronal preoperative CBCT shows that the roots of maxillary second molar were placed directly in the right maxillary sinus. **E** CBCT four months after graft placement shows that the graft has integrated with the bone and that there was sufficient buccal and palatal bone to place two implants. **F** Panoramic image 8 months post-operatively shows two integrated implants with no signs of peri-implantitis

Extraction of teeth 16 and 17 was performed (Fig. 2). Clinical examination after extraction of tooth 17 diagnosed a passage between the extraction site and the right maxillary sinus. The patient was instructed to close the nose with the fingers and blow air through the nostrils, which produced a whistling sound and confirmed the existence of the communication (Valsalva manoeuvre). After thorough debridement of all soft tissues at the extraction site, a magnesium barrier membrane was prepared, inserted, and bent over the bony walls of the extraction site, which rested against the mesial and lateral walls. The magnesium membrane provided a secure separation between the sinus cavity and the extraction site. Both extraction sites were filled with augmentation material and covered with a second magnesium membrane. The flap was released with a subperiosteal incision and sutured with 5–0 nylon sutures.

Four months post-operative, newly formed bone was present, including regenerated buccal and palatal plates with no penetration of graft material into the sinus. At this timepoint, the magnesium membranes had completely resorbed. Two dental implants were implanted at the sites of the extracted teeth.

Eight months post-operative, complete regeneration of the sinus floor at the extraction site can be seen on the panoramic image. The implants have achieved primary stability and the soft tissue shows desirable healing.

### Case 2

The patient was a 54-year-old female in good general health. The patient presented with endodontically treated tooth 16 and an old porcelain-fused-to-metal (PFM) crown. The apical ends of the palatal, mesiobuccal, and distobuccal roots penetrated inside the right maxillary sinus.

**Fig. 3:**
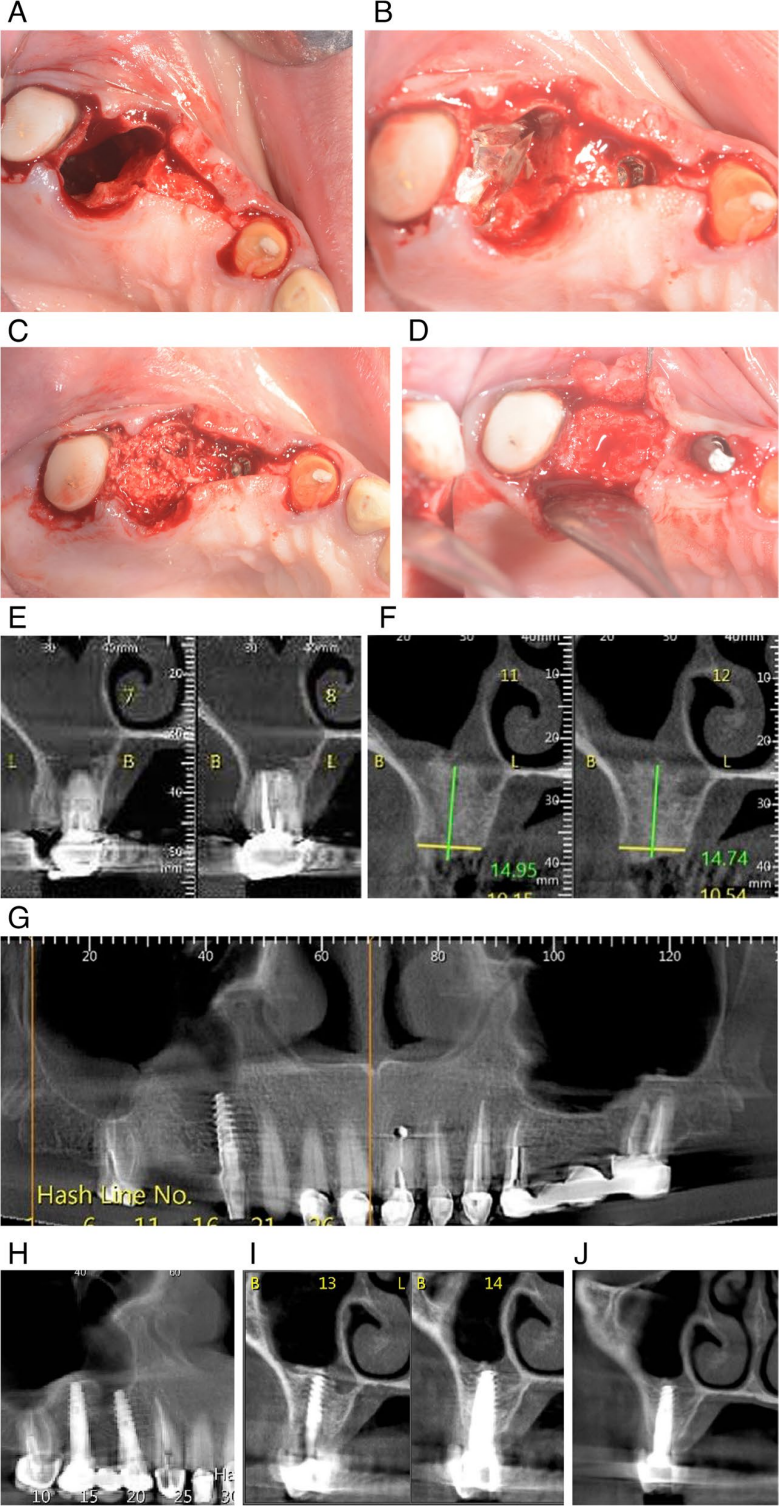
**A** Tooth 16 was extracted, leaving communication between the oral cavity and the maxillary sinus. **B** The sulcus was opened through a crestal incision and the dental implant was placed at position 14 (blue arrow). Resorbable magnesium membrane was shaped to close the communication at position 16 (black arrow). **C** The cavity at position 16 was filled with allograft and xenograft granules, the soft tissue was sutured, and a healing cap was placed on the implant (blue arrow). **D** Five months post-operatively, the implant was stable, with no abnormal movement, and the graft material was integrated with surrounding healthy hard tissue. **E** Coronal CBCT section taken before extraction shows that there is no alveolar bone between the apical third of tooth 16, which ends in the right maxillary sinus. **F** CBCT cross-section taken five months after graft placement shows 15 mm of newly formed bone, sufficient for placement of a new fixed restoration. **G** Panoramic image taken five months after implant placement at position 14. Newly formed bone tissue has formed adjacent to the implant with no signs of peri-implantitis. **H** The panoramic image was taken four months after implant placement at position 16 in the maxilla. A dental bridge is attached to implants 14 and 16 as well as healthy tissue around the previously placed implants. **I**, **J** Coronal CBCT images slices show correct placement of the implant between the buccal and palatal alveolar walls and the presence of new alveolar bone in the apical third of the implant, creating complete separation from the right maxillary sinus

Because the apical ends of tooth 16 were located in the sinus, slow and atraumatic extraction of the tooth was performed. After clinical examination, communication between the right maxillary sinus and the oral cavity was established (Fig. 3). A crestal incision was made between teeth 13 and 17. A magnesium membrane was shaped and used to close the communication. In this case, the membrane supported the opposing alveolar walls to close the gap. The extraction site was filled with allograft and xenograft granules. The dental implant was placed at position 14, achieving primary stability.

Five months post-operatively, an implant was placed at position 16, achieving primary stability. Upon opening the flap, there was no remnants of the magnesium membrane and satisfactory vertical and horizontal bone gain had been achieved.

### Case 3

The patient was a 51-year-old female in good general health. The patient presented with tooth 26, which was used as an abutment for a dental bridge. The said tooth had ¼ of horizontal bone loss, showed horizontal and vertical mobility and its roots ended in the left maxillary sinus.

**Fig. 4:**
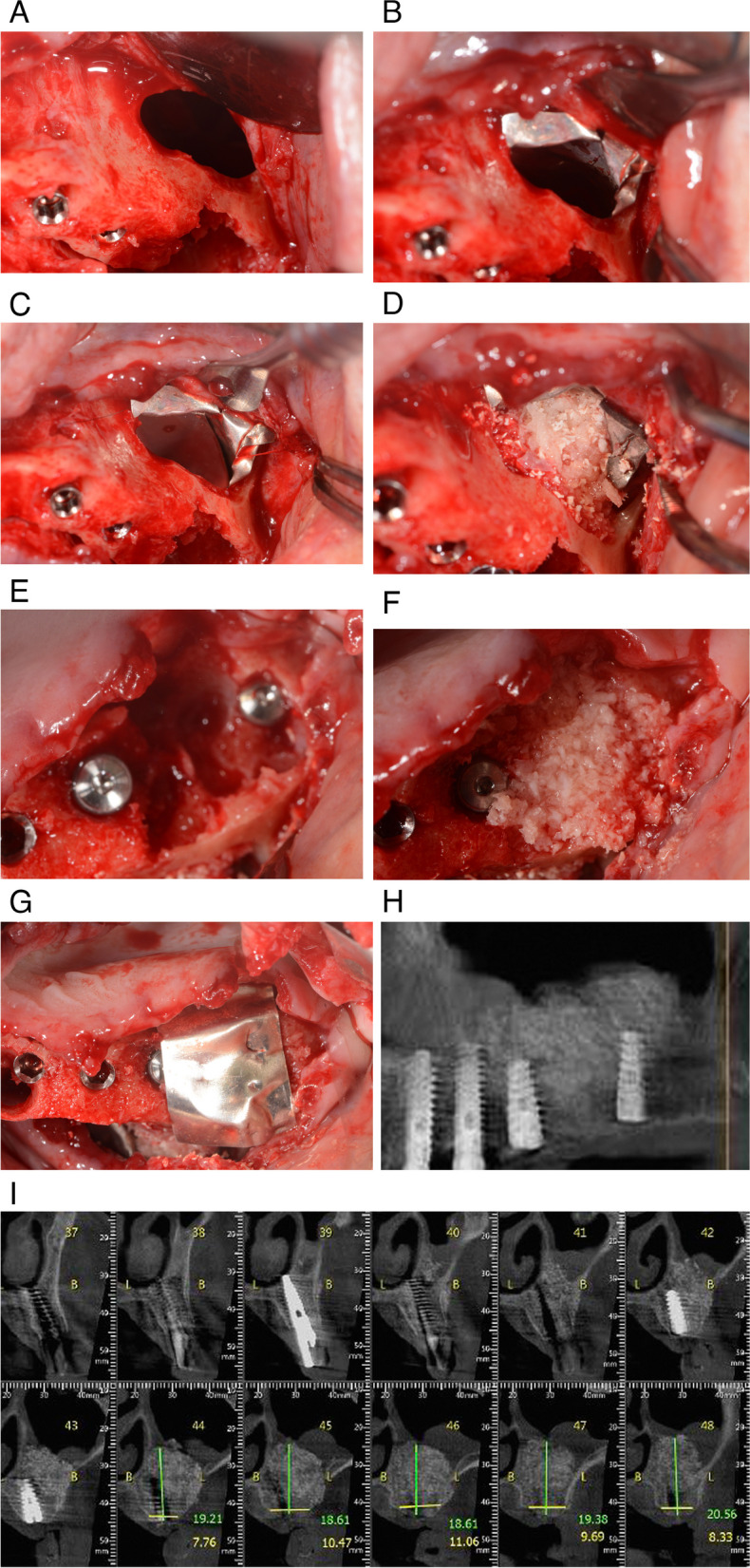
**A** Extraction of tooth 26 resulted in a large loss of bone tissue. A direct approach was used to open the buccal wall to facilitate access to oroantral communication (black arrow). **B** A magnesium membrane was placed over the buccal window to close the connection between the maxillary sinus and the extraction site (blue arrow). **C** Second layer of membrane is applied on top of the first layer (blue arrow). **D** The site was augmented using allograft and xenograft granules. **E** Four dental implants were immediately placed. **F** Allograft and xenograft granules were also used to augment the extraction site. **G** A magnesium membrane was used as a barrier to create space between bone and soft tissue (blue arrow). **H** Panoramic image four months after placement of four implants at positions 23, 24, 25, and 27 and application of graft material shows stability and formation of new alveolar bone. **I** Coronal CBCT image shows 18–20.5 mm of alveolar bone between the oral cavity and the floor of the left maxillary sinus

After extraction of tooth 26, the Schneiderian membrane remained intact. In this case, an open sinus lift was performed. The buccal window was surgically opened with a round burr at low speed to gain easier access to the floor of the sinus. During this step, the Schneiderian membrane completely ruptured, creating a communication between the sinus and the oral cavity (Fig. 4). The magnesium membrane was cut to fit the shape and then placed to close the communication. Allograft and xenograft granules were placed on top of membrane to close the buccal window and rebuild the buccal wall.

A rigid magnesium membrane was molded to close the buccal window and support the augmentation. Four implants were placed immediately at positions 23, 24, 25 and 27.

Four months post-operatively, excellent regeneration and stability were evident on the panoramic image. CBCT measurements indicate 18 mm of newly formed alveolar bone was gained between the sinus and the oral cavity.

### Case 4

The patient was a 61-year-old male in good general health. The patient presented with two dental implants at positions 14 and 16 and a fixed dental bridge on top of them. Palpation of the bridge revealed movement in both the vertical and horizontal dimensions. Panoramic images taken prior to surgery showed evidence of peri-implantitis with vertical bone loss extending to half the height of implant 14, while implant 16 was placed in the right maxillary sinus and surrounded by a large polyp.

**Fig. 5:**
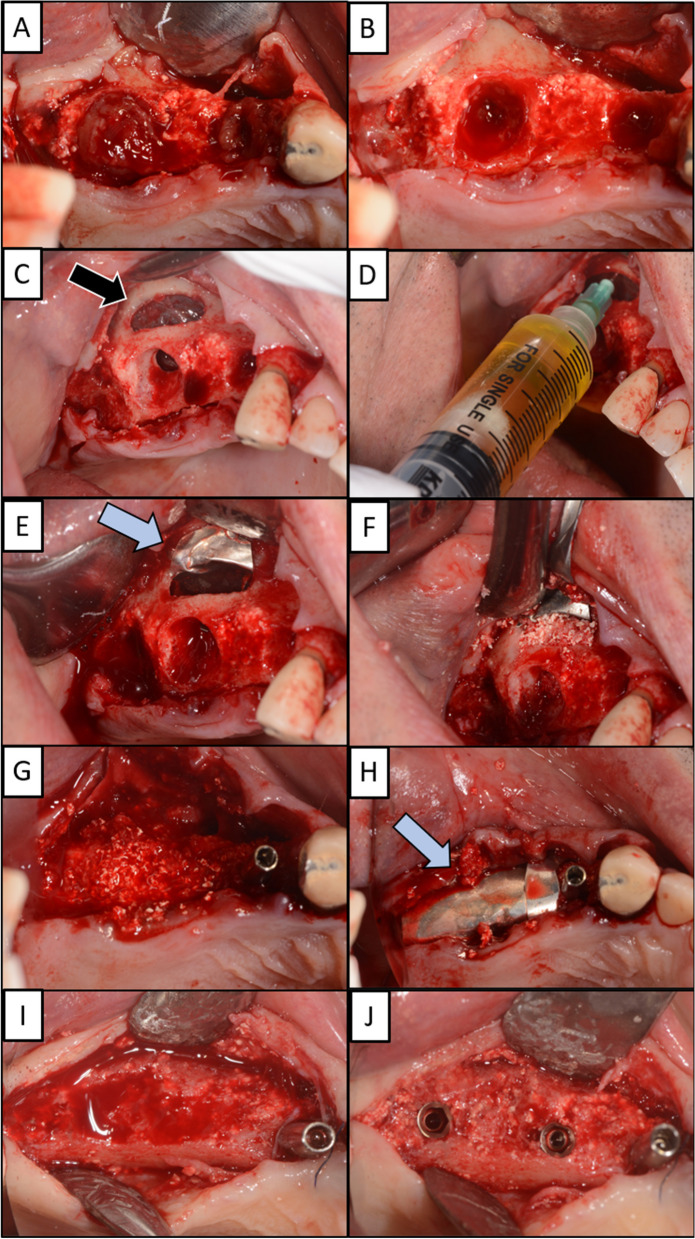
**A** Extraction of dental implants at position 14 and 16 resulting in large bone defects. **B** After removal of the additional soft tissue at the extraction site, there is a better view of the oroantral communication. **C** The window on the buccal wall was surgically opened to gain direct access to the Schneiderian membrane (black arrow). **D** Aspiration needle was used to aspirate the polyp inside the right maxillary sinus through the Schneiderian membrane. **E** A magnesium membrane (blue arrow) is placed on Schneiderian membrane to stimulate and form the separation of the oral cavity from the right maxillary sinus. **F** The buccal window is closed with allograft and xenograft granules. **G** Immediately after extraction, an implant is placed in position 14, the site of the previous implant, since a greater amount of alveolar bone is preserved here compared to position 16. **H** A magnesium membrane (blue arrow) is placed as a barrier on extraction site 16 to cover the graft and separate it from the soft tissue. The soft tissue is sutured to achieve primary healing. **I** Five months later, the magnesium membrane has resorbed and new hard tissue has formed at site 16, which can be used to place new dental implants. **J** Two dental implants are placed at position 15 and 17

Atraumatic extraction of dental implants 14 and 16 was performed (Fig. 5). Schneiderian membrane remained complete. Direct sinus lift approach was instrumented to gain access to the maxillary sinus floor. The buccal window was surgically opened with a round burr. Using aspiration needle, polyp in the right maxillary sinus was drained and removed. Magnesium membrane was used in a similar manner to the previous case and rested on alveolar bone of buccal window. The allograft and xenograft granules were packed tightly into the buccal window to seal this portion from the remaining alveolar bone. Additional allograft and xenograft material was placed in the extraction site and covered with a long piece of magnesium membrane along the length of the alveolar ridge to provide adequate coverage and separation from the soft tissue. A dental implant was placed at position 14.

**Fig. 6:**
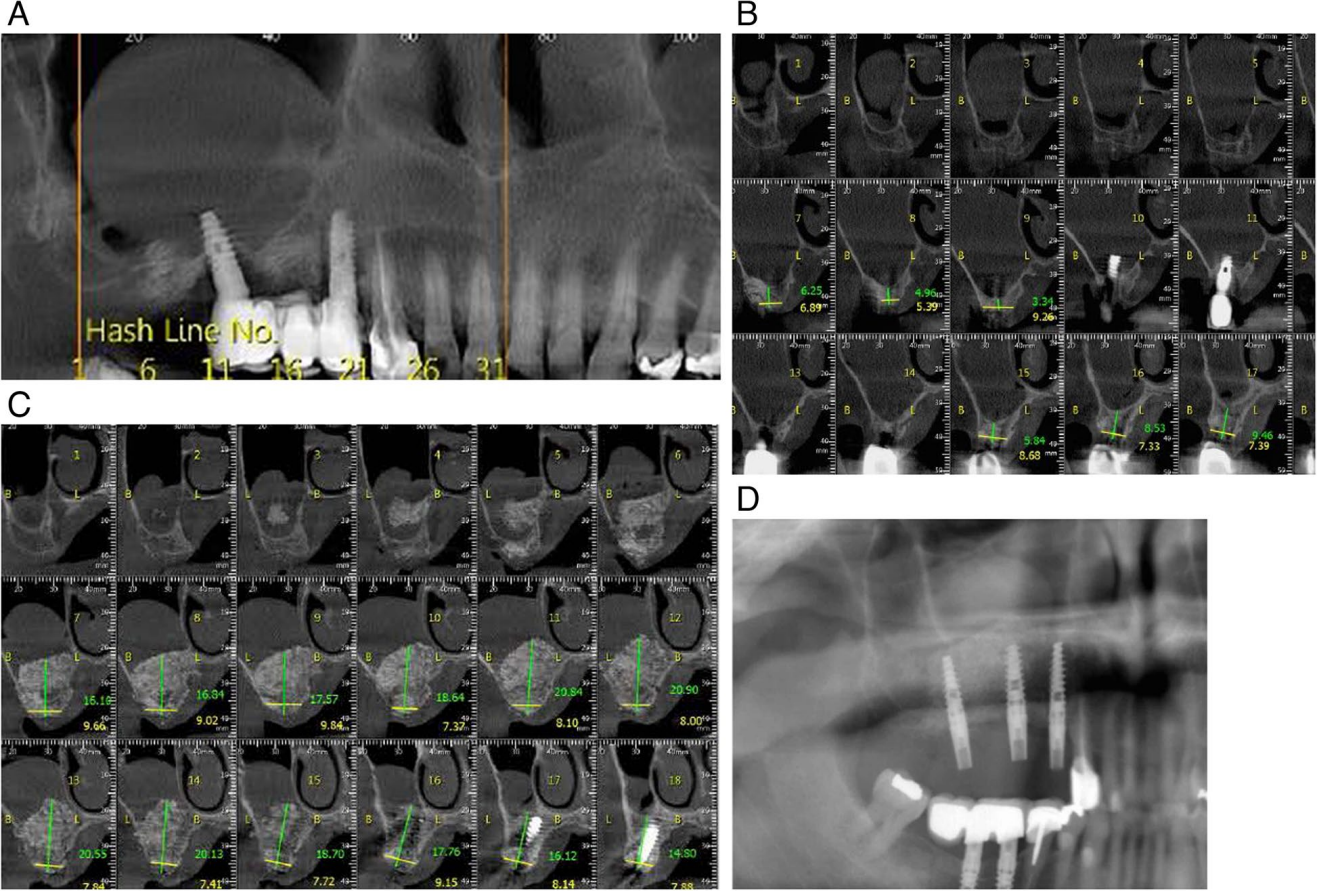
**A** The dental implants were positioned in the maxillary arch at position 14 and 16. The panoramic image before extraction shows polyp in the right maxillary sinus and peri-implantitis surrounding the implant. Both implants are indicated for removal due to their decreased stability and vertical and horizontal mobility at stage 3. **B** Coronal CBCT section shows the implants in the maxillary sinus with a minimal alveolar bone level of 3.5–9 mm in the vertical dimension. **C** The coronal CBCT section after graft placement shows that new bone has formed over a period of five months. **D** The panoramic image shows that the three new implants are seated in the alveolar bone at positions 14, 15, and 17 and demonstrate primary stability on clinical examination

Five months after surgery, an incision was made along the alveolar crest to inspect the bone level. The magnesium membrane covering the defect had completely resorbed. The bone exhibited optimal hardness and composure. The CBCT image displayed 16–21 mm of newly formed bone, which varied at different locations (Fig. 6). Two additional dental implants were placed at position 15 and 17.

## Discussion

This case series showed that magnesium membrane can be useful in the repair and regeneration of Schneiderian membrane perforation that occurs during a sinus lift performed directly or indirectly. Although the sinus lift procedure is well established, complications are common. Perforation of the Schneiderian membrane can occur in up to 42% of cases [18] and is caused by anatomic variations such as reduced thickness of the membrane, or via iatrogenic conditions such as by an inexperienced operator or incorrect instrument manipulations [20]. The Schneiderian membrane has an average thickness of 0.79 ± 0.52 mm as measured by cone beam computed tomography [32]. The membrane becomes thicker at the level of the periosteal layer, where it tends to be elastic and stretchable, making it difficult to accidentally perforate. However, inflammation can induce a thickening of the membrane, causing it to become gelatinous and prone to perforation once the surgeon has removed the periosteal layer [33, 34].

Treatment options vary depending on the extent of membrane perforation. Published studies indicate that for perforations less than 5 mm in diameter, suturing and the application of a resorbable collagen membrane, demineralized laminar bone membrane, or fibrin glue are among the possible treatment options [25]. In some instances, if the perforations are very small in diameter, the perforations can heal without further intervention as the membrane folds over itself as the elevation progresses [35]. When the perforation is larger than 5 mm, the use of graft material and a barrier membrane is indicated [25, 36, 37]. According to the literature [38], mixing two different graft materials (allograft and xenograft) reduces the loss of vertical and horizontal dimensions after tooth extraction compared to using only allograft material in grafting procedure. The xenograft is usually composed of pure hydroxyapatite, which is only slightly resorbed overtime and is instead incorporated into newly formed bone. Therefore, the xenograft provides long term volume stability to the augmentation. Proussaefs and Khoury [36, 39], have reported that rupture of the membrane during surgery decreased graft survival in half of their cases. Other studies concluded that properly repaired perforations have no effect on dental implant survival [40].

Resorbable collagen membranes are a reliable material to support the healing of the Schneiderian membrane due to their biocompatibility, potential to promote wound healing, and the fact that no second surgical procedure is required for removal as they degrade and are replaced by the surrounding tissue [41]. However, for large perforations, collagen membranes have limited stiffness and cannot resist the pressures applied by the surrounding soft tissue [19]. Additionally, rapid biodegradation, can result in unsatisfactory clinical outcomes and an unpredictable bone regeneration [42]. In instances of complete tears of the Schneiderian membrane, it is often recommended to abort the augmentation procedure until a later date after the membrane has repaired itself [21, 39].

The recent development of a magnesium metal membrane could provide the solution for the treatment of large perforations as well as tears of the Schneiderian membrane. Magnesium-based metals have been investigated for medical applications for over a century [43], but have only recently found success as orthopaedic screws and cardiovascular stents [44, 45]. More recently, the development of a magnesium membrane and fixation screws opened up new possibilities in dentistry [29, 46–48]. The magnesium membrane, which is used in the reported case series, is rigid as well as highly biocompatible and completely degradable [46, 49–51]. Magnesium ions, released during the degradation of magnesium metal, are naturally present within the body and are involved in many important processes, such as mitochondrial activity, protein and DNA synthesis, and blood pressure regulation [52–54].

In a study by Rider et al. [29], it was reported that when magnesium degraded, the pH of the surrounding tissue environment becomes more alkaline, which may prove to have an anti-inflammatory effect [55]. Another finding of this study is that magnesium membranes have a much higher tensile strength (183.0 ± 10.7 MPa) than that of collagen membranes (4.8 to 22.5 MPa). The additional mechanical support provided by the magnesium membrane could be beneficial for providing support for large perforations of the Schneiderian membrane.

According to Rider et. Al. [46] in their animal studies on beagle dogs, the membrane undergoes significant degradation between 1 and 8 weeks after onset, lasting until 16 weeks. In vitro studies per Rider et. Al. [29] imply that magnesium salts retain the original shape and position of the magnesium metal after 1, 2, and 4 weeks until they are absorbed. Complete bioresorption of the magnesium membrane is reported at 16 weeks. Another advantageous feature of the magnesium membrane is its ductility, which accounts for the quick shaping during surgery [49, 50]. It has also been demonstrated that human fibroblastic cells will adhere and migrate over the surface of the membrane, potentially aiding the healing of the Schneiderian membrane [56]. The combined properties enable the magnesium membrane to create a stable new roof for the augmentation, maintain a separation of the augmentation from the sinus, as well as have a positive interaction with the repair of the Schneiderian membrane. As a byproduct of the degradation process of magnesium metal, hydrogen gas is released. This is clinically visible as small gas pockets that usually form above the membrane and can present as a slight swelling of the soft tissue. In a previous study, Rider et al. showed that these pockets spontaneously resolve after the magnesium metal has degraded and had no negative effects on the regenerative outcome in comparison to sites treated with a collagen membrane in Beagle dogs [46]. The gas pockets could have a positive effect by serving as an additional barrier between the soft tissues of the maxillary sinus and the hard tissues of the alveolar bone [49].

In this paper, a case series consisting of four cases is presented and describes the application of a magnesium membrane as a method of separating the maxillary sinus during sinus lift procedures where there has been a perforation of the Schneiderian membrane.

In the first and second case, a small to moderate perforation, approximately 5 mm in diameter, had occurred. The magnesium membrane was shaped to close the oroantral communication in a vertical approach within the defect, and was supported by the mesial and lateral walls. This approach avoided unnecessary damage to the surrounding tissue caused by opening an additional surgical site for access to the sinus. A second magnesium membrane was placed on top of the graft and was used to separate the graft material from the soft tissue. This type of reconstruction provided sufficient mechanical support and strength to the augmentation to support the regeneration process.

The third case had large perforation of the Schneiderian membrane caused during the opening of a second surgical field on the buccal wall. The application of a magnesium membrane for the treatment of large perforations instead of collagen membranes could potentially lead to more successful healing as they can provide greater stability. In this case, a large bone volume was successfully gained. In the fourth case, the magnesium membrane was also applied in a direct approach, however in this case, the Schneiderian membrane remained intact. Again, a large bone volume was successfully achieved.

Before their application, each membrane had to be adapted to the particular defect. In the first and second cases, the magnesium membranes were conically shaped to completely close the defect and the sides of the membrane rested on the mesial and distal walls of the alveolar bone. In the third and fourth cases, where the ruptures were much larger, large pieces of membrane had to be used. In the fourth case, the membrane spread from the defect through the buccal window and rested against the buccal surface of the alveolar bone, providing support for the graft material used to fill large bone defects.

Because of the material's ability to be malleable but stable, shaping the magnesium membranes during surgery to the appropriate dimensions and curves of the sinus opening was quick and easy. Postoperative consultation revealed healthy, newly formed bone in all cases, which was confirmed by panoramic images and CBCT.

Overall, the application of a magnesium membrane in sinus lift augmentations and to treat perforations and complete rupture of the Schneiderian membrane, has proven to be successful in both indirect and direct approaches. In large defects it has been shown to be an ideal material due to its stiff but ductile properties that enable an ease of application whilst also supporting the augmentation. Additionally, it is completely resorbable and does not need to be removed in a second surgery.

The presented case series showed positive results. In order to draw further conclusions about the mentioned properties of this material, the application of resorbable magnesium membrane in different situations and over a longer period of time with comparison to control group is necessary.

## Conclusions

For the first time in regenerative dentistry, the use of magnesium membranes in direct and indirect sinus lift procedures and their results have been documented. Collagen membranes represent the standard material of choice for repair of the Schneiderian membrane. However, for larger perforations and tears of the Schneiderian membrane, a magnesium membrane has been presented as an alternative material choice as it offers the stiffness of metal, whilst being completely resorbable. The case series performed provided satisfactory results with successful healing in four cases and formation of new cortical bone, complete separation of the maxillary sinus from the oral cavity, and closure of oroantral communication. Considering that this is the first reported use of resorbable magnesium membrane in sinus lift procedures, further clinical studies and a larger number of cases would help to confirm the facts presented in this case series and its use in regenerative dental procedures in the next decade.

## Data Availability

The data presented in this article are available on request from the corresponding author.
